# Syntheses of Isoxazoline-Carbocyclic Nucleosides and Their Antiviral Evaluation: A Standard Protocol

**DOI:** 10.1155/2014/492178

**Published:** 2014-10-30

**Authors:** Paolo Quadrelli, Naiara Vazquez Martinez, Roberto Scrocchi, Antonino Corsaro, Venerando Pistarà

**Affiliations:** ^1^Dipartimento di Chimica, Università degli Studi di Pavia, Viale Taramelli 12, 27100 Pavia, Italy; ^2^Dipartimento di Scienze del Farmaco, Università degli Studi di Catania, Viale A. Doria 8, 95125 Catania, Italy

## Abstract

The current synthesis of racemic purine and pyrimidine isoxazoline-carbocyclic nucleosides is reported, detailing the key-steps for standard and reliable preparations. Improved yields were obtained by the proper tuning of the single synthetic steps, opening the way for the preparation of a variety of novel compounds. Some of the obtained compounds were also evaluated against a wide variety of DNA and RNA viruses including HIV. No specific antiviral activity was observed in the cases at hand. Novel compounds were prepared for future biological tests.

## 1. Introduction

Carbocyclic nucleosides (carbanucleosides) [1] are modified nucleosides [2] in which a methylene group has replaced the oxygen atom of the furanose ring [3]. Due to the absence of a glycosidic linkage, carbanucleosides are chemically more stable and not subjected to the phosphorylases that cleave the* N*-glycosidic linkage in conventional nucleosides [4]. Many compounds of this class with potent and selective biological activity have been identified [5], including carbovir [6]** 1** and abacavir [7]** 2**, which are currently used therapeutically as anti-HIV agents.

**Figure 1:**
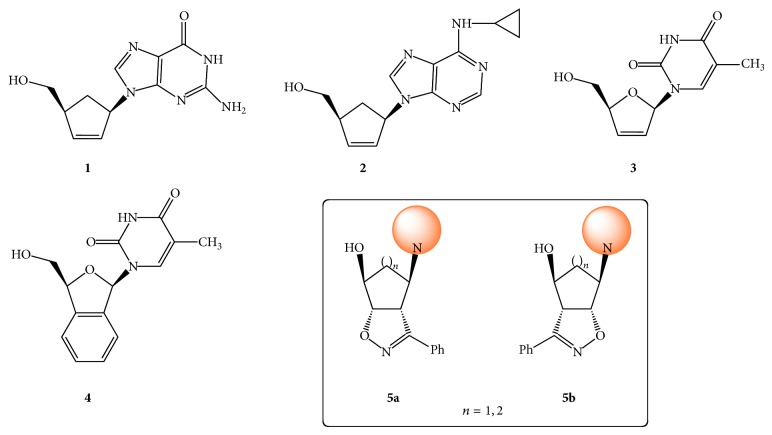


An important aspect of the anti-HIV therapy is the suppression of viral replication in the brain; in this regard an enhanced lipophilicity of potential drugs is likely to be advantageous. Recently, Vince and Hua [6] have reported the modification of the stavudine** 3** by fusion of a benzene ring to the carbasugar, leading to analogue** 4** [8]. This structural modification increases the lipophilicity (and hence the access to the central nervous system, a major reservoir of HIV) [9] while maintaining the rigidity imposed by the C2′–C3′ double bond. Analogously, carbanucleosides in which an aromatic heterocycle is fused to the carbasugar of carbovir and abacavir have been prepared [10]. Five-membered heterocyclic rings such as a pyrazole [11] or isoxazoline [12] are often found in carbocyclic nucleosides [13] as expedients to tune up the lipophilicity or reduce the conformational flexibility of the carbocyclic moiety (Figure 1) [14].

Recently, we have reported the synthesis of carbocyclic nucleosides** 5** containing a fused isoxazoline ring and lacking a methylene (CH_2_) group in the side chain in the carbocyclic unit [12–14]. Nucleosides lacking a methylene group in the side chain have been reported and in some cases display reduced cytotoxicity [15]. The synthetic route that we have exploited, based on the hetero-Diels-Alder (HDA) cycloaddition of dienes with transient acyl nitroso moieties [16], affords “privileged structures” [17] for the synthesis of carbocyclic nucleosides [18].

However, some steps of the synthetic procedure for the linear construction of the nucleobases were problematic and not all of the synthesized compounds could be prepared on a suitable scale for biological tests. Because of these problems, at the beginning of our work, it was not possible to fully evaluate the biological activity of this class of nucleosides, and further structural modifications, necessary for an improved antiviral activity and for a better understanding of the structure-activity relationship (SAR), were sometimes forbidden.

On pursuing our studies on the biological activity of carbanucleosides, we present here the improved synthetic procedure of compounds of type** 5 **(*n* = 1), amenable to a larger scale preparation and for the preparation of a variety of novel compounds. Their inhibitory activity against a wide range of DNA and RNA viruses, including HIV, is also reported and discussed.

## 2. Results and Discussion

### 2.1. Chemistry

**Scheme 1:**
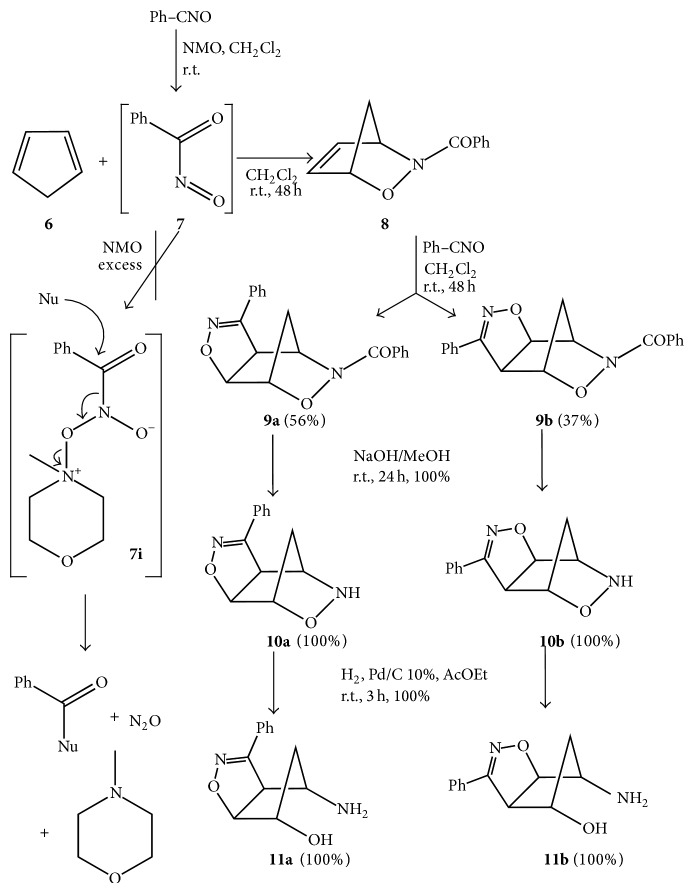


The HDA cycloadduct* N*-benzoyl-2,3-oxazanorborn-5-ene** 8** was obtained from freshly distilled cyclopentadiene** 6** (2 equivalents) that quantitatively trapped the nitrosocarbonyl benzene** 7** generated* in situ* through the mild oxidation of benzonitrile oxide Ph-CNO (BNO) in CH_2_Cl_2_ with a slight excess (1.3 equivalents) of* N*-methyl-morpholine* N*-oxide (NMO) according to the published procedure (Scheme 1) [18, 19] in good yields (73%). It is essential not to exceed with the NMO amounts in this preparation because a larger excess of the oxidant could be detrimental for the nitrosocarbonyl intermediate's life, since the* N*-oxide could add the intermediate and promote its decomposition [16, 18, 19]. In Scheme 1 the decomposition pathway is sketched. Nitrosocarbonyls are in fact short-living species, just 180 *μ*s as reported by Cohen and coworkers [20] and, if not instantly trapped, can add the excess NMO to give the intermediate** 7i** that evolves to add nucleophilic species which can be present in the reaction mixtures even in small amounts to afford* N*-methylmorfoline, N_2_O, and the adduct between the benzoyl group and the nucleophile* Nu* (in case of water, benzoic acid) [21].

The* N*-benzoyl-2,3-oxazanorborn-5-ene** 8** was found to be an excellent dipolarophile towards BNO addition, affording the two regioisomeric cycloadducts** 9a** and** 9b** which were easily isolated in quantitative yields by chromatographic separation in a 3 : 2 regioisomeric ratio [18, 19]. A proper tuning of the subsequent synthetic steps had to be done for the scale-up necessary for the synthesis of nucleosides in the due amount for the biological tests.

The ease detachment of the benzoyl group from the cycloadducts** 9a**,**b** was achieved through NaOH/MeOH treatment at room temperature for 24 h, affording the fairly stable hydroxylamines** 10a**,**b**. The careful revision of the alkaline hydrolysis allowed for improved protocols for this step that was forced to take place in quantitative yields (+20–30% with respect to the previous method) [19]. The reaction has been conducted by adding a slight excess (1.2 equivalents) of NaOH, added portionwise in 2-3 h, to a methanol solution of cycloadducts** 9a**,**b**, stirred at room temperature until complete disappearance of the starting materials. Extended reaction times (from 12 to 24 h) ensured the quantitative transformation of the HDA cycloadducts** 9a**,**b** into the desired hydroxylamines** 10a**,**b**. These compounds were previously fully characterized [19] and found completely stable in the solid phase but they do not survive for long when left in solution and decompose in several uncharacterized products in miserable yields.

The hydrogenolytic cleavage of the N–O bond of the hydroxylamine** 10a**,**b** has been also improved and takes place also quantitatively (+30–40% with respect to the previous method) [19] by performing the reaction with Pd/C 10% in ethyl acetate at room temperature for 3 h (extended time). Hence, the aminols** 11a**,**b** were obtained and found identical with authentic samples previously prepared [19]. The regioisomeric aminols** 11a**,**b** were then submitted to the transformations into the desired nor-nucleosides [13] through linear construction of the purine and pyrimidine rings [22].

**Scheme 2:**
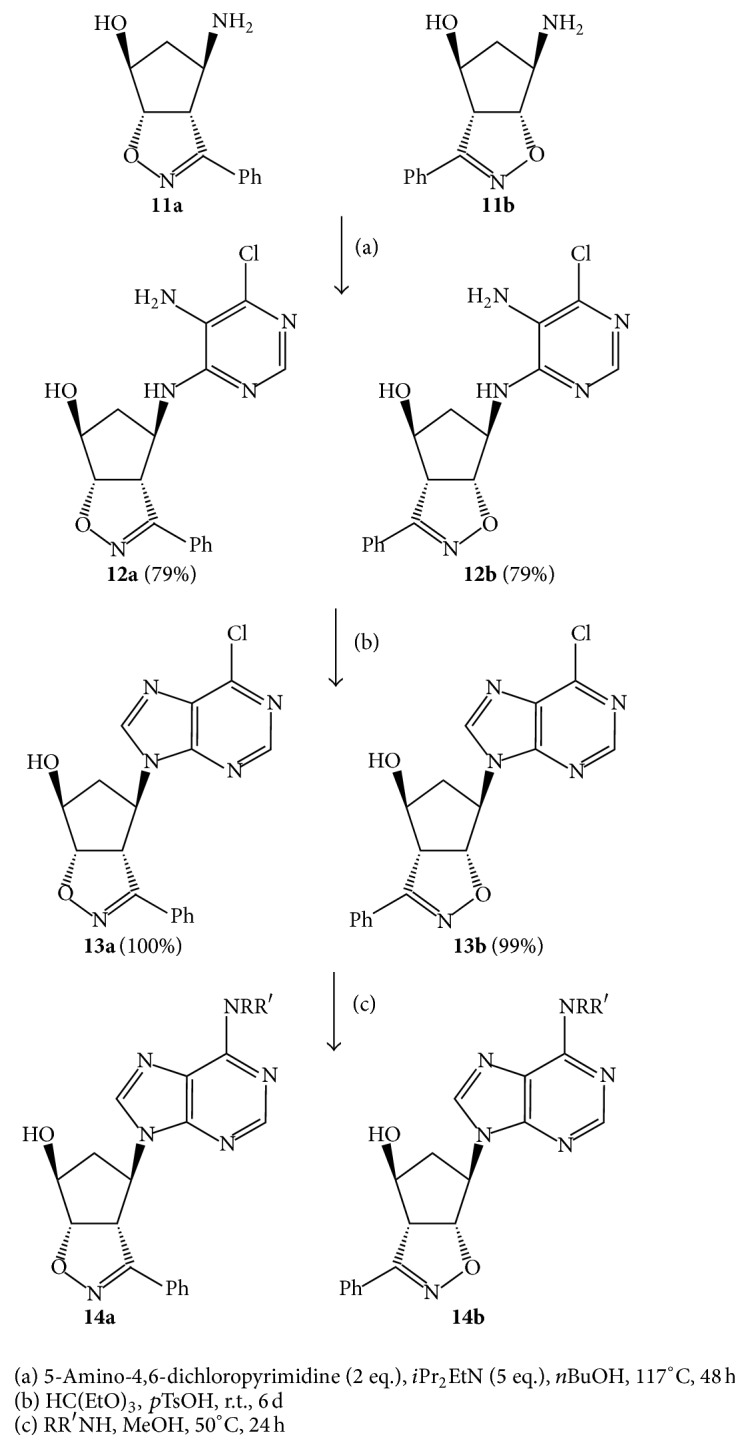


The synthesis of the purine-nucleosides** 13** requires the two steps sketched in the Scheme 2.

The first one is the condensation of the regioisomeric aminols** 11a**,**b** with the 5-amino-4,6-dichloropyrimidine to give the pyrimidine derivatives of type** 12a**,**b**. This condensation is indeed the most difficult step in the synthesis, which attained only low yields (around 50%) in the previously reported preparations [13]. Better yields of** 12a**,**b** were achieved by increasing the reaction temperature gradually up to the boiling point of the solvent. An excess (2 equivalents) of the 5-amino-4,6-dichloropyrimidine was added to a* n*-BuOH solution of the aminols** 11a**,**b** in the presence of 5 equivalents of* i*-Pr_2_EtN. The solutions are stirred at room temperature for 1 h and a gradual increase of temperature at the boiling point of* n*-BuOH (117°C) was conducted in 1 h in sealed tubes. After 48 h under these conditions the* n*-BuOH was removed at reduced pressure and the residues were taken up with dichloromethane (DCM) and washed with water. The residues, obtained by evaporation of the DCM, were submitted to chromatographic separation to isolate, from the excess of reagent, the pyrimidine derivatives** 12a**,**b** in 79% yields for both regioisomeric compounds. Any attempt to shorten the reaction time by using microwave irradiation instead of conventional heating unfortunately failed. The structures of compounds** 12a**,**b** were confirmed through comparison with authentic samples and spectroscopic analyses. For NMR details we send back to [13].

The conversion of the pyrimidine derivatives** 12a**,**b** into the chloropurine compounds** 13a**,**b**, which was found previously somewhat problematic due to incomplete ring closure and instability of final products [13], has been also simplified.

Solutions of** 12a**,**b** in ethyl orthoformate were stirred at room temperature until the pyrimidine derivatives were completely converted into the formylated compounds (monitoring by TLC, 5–7 h) and at this time a catalytic amount of* p*TsOH was added and stirring continued for 5 days. On applying the frequently reported methods [23] using triethyl orthoformate in the presence of 37% HCl at r.t., no condensation took place and the starting materials were recovered unchanged after the suggested work-up. Harsher conditions [24] sometimes allow for the obtaining of the desired product albeit in poor yields, but in most of the cases the starting material decomposed. The rather tedious work-up procedure (orthoformate evaporation, addition of Et_3_N to a CHCl_3_ solution and stirring for 24 h, washing with water and drying, evaporation of the CHCl_3_, residues taken up with EtOAc and stirring for several hours, evaporation of the solvent, and chromatographic separation) has been simplified by adding water to the orthoformate solutions to hydrolyze the excess orthoformate and the formylated intermediates and allowing the solution to stand at room temperature for 24 h. Extraction with DCM allowed isolating in quantitative yields (+10%) the chloropurine derivatives** 13a**,**b** which have been characterized spectroscopically and found identical with authentic samples (see [13] for NMR details).

**Figure 2:**
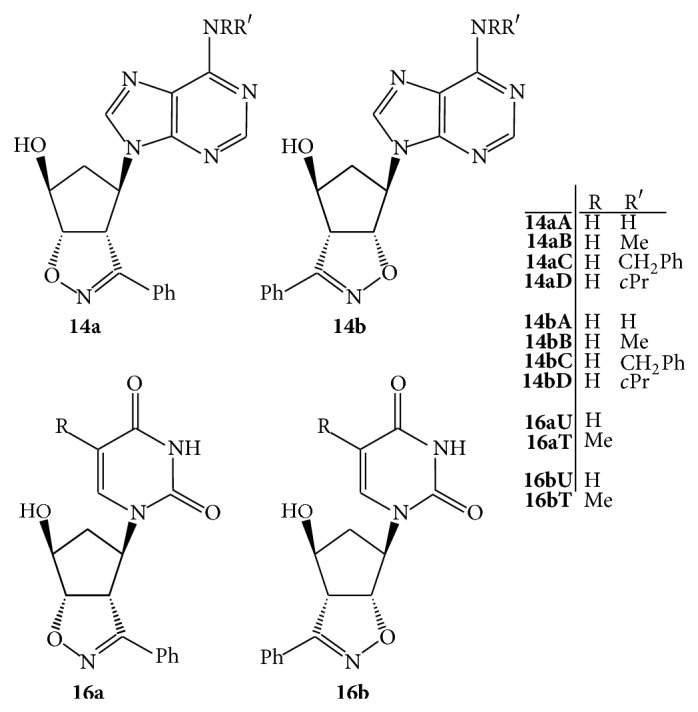


The final chlorine replacement method with selected amines did not require any revision since the substitution occurs with high yields and was conducted as previously described [13] upon heating MeOH solutions of** 13a**,**b** at 50°C in the presence of an excess NH_3_ or some primary amines (methyl-, benzyl-, and cyclopropyl-amine) affording the adenine derivatives** 14a**,**b** in more than 92% yields (HPLC) which have been submitted to the biological tests (Figure 2).

**Scheme 3:**
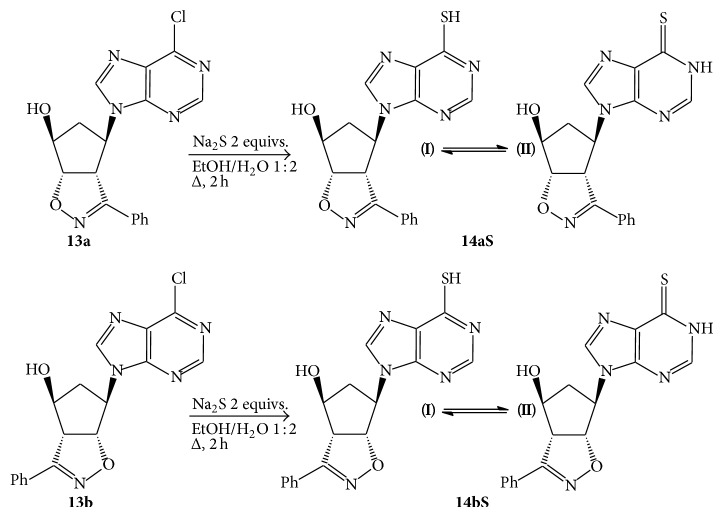


However, this type of derivatization is not applicable to all the groups to be inserted as nucleophiles. Alcoholic solvents remain the base to dissolve properly the chlorine-nucleosides but the presence of a cosolvent has to be evaluated, accordingly [25]. This is the case of the insertion of a thio-group (SH) in place of the chlorine atom (Scheme 3).

The chlorine-nucleosides** 13a**,**b** were dissolved in an hydroalcoholic solution in the presence of excess Na_2_S and the mixture was heated at reflux for a couple of hours. Upon neutralization with acetic acid 10% and cooling, the thio-derivatives** 14a**,**bS** crystallized and were easily filtrated (**14a**,**bS**, 89% and 87% yields, resp.). Their structures rely upon the corresponding analytical and spectroscopic data and this characterization evidenced the presence of the tautomeric equilibrium between the two forms reported in Scheme 3.

The tautomeric ratio varied upon the polarity of the solvent and Table 1 reports the ratios determined in representative polar solvents and the chemical shifts of the main protons of the structures reported. The ratios were determined through NMR spectroscopic studies by measuring the integral ratios of the CH=N sharp singlets in the solvents reported and the attribution to the two tautomers of the different groups of signals was done upon COSY experiments.

Upon comparison of the NMR spectroscopic data of compounds** 14a**,**bS** and the NMR data reported in literature [26], it was possible to attribute unequivocally the structures of the two tautomeric forms for both the regioisomers [27]. It is known in fact that the thioxo form** II** is greatly stabilized in aqueous solution [28]. The NMR data in our hands suggest that the form** II** is the major component in some of the mixtures and the ratios can be reverted by changing the solvent. The equilibrium is slow and, besides the aromatic signals of the phenyl groups, which are coincident, most of the other signals both from the purine heterocyclic ring and the isoxazoline-cyclopentanol moiety differ remarkably allowing for the clear resolution of the two tautomers.

A different behaviour is noticeable between the two regioisomers** 14aS** and** 14bS**. In deuterated acetone, regioisomer** 14aS** is mainly present as the thioxo tautomer** II** whose proton signals are downfield shifted with respect to tautomer** I** in nice keeping with the observations reported in literature for thiopurine derivatives [28]. The two tautomers equally coexist in deuterated methanol while in DMSO the ratio is remarkably reverted in favour of the mercapto form** I**. This is presumably due to the known ability of DMSO to establish H-bonds, in this case with the SH group, hence stabilizing the tautomer** I**.

The second regioisomer** 14bS** shows a diminished variation of the tautomeric ratio as a function of the solvent changes. Both in DMSO and deuterated methanol the ratios are close to 1/1. Only in acetone tautomer** I** is twice the other.

The stereoisomeric aminols** 11a**,**b** were also converted into the uracil and thymine nucleosides. Among the several methods reported in literature for the synthesis of pyrimidines [29] and in particular those involving the formation of two bonds suitable for uracils preparation, the linear construction of these heterocycles [30] through condensation of aminols** 11a**,**b** with the appropriate isocyanates was preferred because of its wide applications in a variety of substrates and experimental conditions as well as for the excellent results achieved [31].

**Scheme 4:**
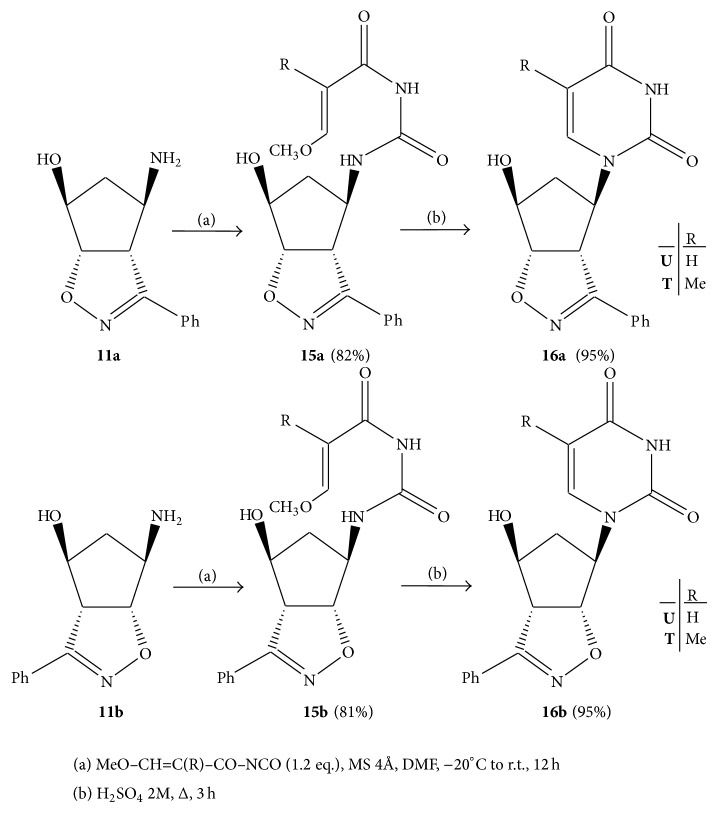


The synthetic route to uracil and thymine nucleosides involves the steps illustrated in Scheme 4 and started with the preliminary preparation of the isocyanates whose quality and purity strongly determine the yields of the final compounds. The condensations of the isocyanates with aminols** 11a**,**b** were performed at –20°C in anhydrous DMF solutions and the reactions left at room temperature for 12 h. The urea adducts** 15a**,**b** were obtained in 82% yields and found identical with authentic samples previously prepared [13]. Cyclization of the ureas** 15a**,**b** took place smoothly upon gentle refluxing in 2 M H_2_SO_4_ solution for 3 h. The uracil nucleosides** 16Ua**,**b** and the thymine analogues** 16Ta**,**b** were isolated from these solutions after pH adjustment to** 7** and extraction with dichloromethane. The yields of the cyclization steps were satisfactorily improved (95%, +20%) and the structures confirmed through spectroscopic analyses.

The products were submitted to biological evaluation (Figure 2).

### 2.2. Antiviral Activity

Compounds** 14a**,**b** and** 16a**,**b** were evaluated for their inhibitory activity against a wide variety of viruses, including herpes simplex virus type 1 (HSV-1 (strain KOS) and 2 HSV-2 (G)), vaccinia virus (VV), vesicular stomatitis virus (VSV), herpes simplex virus 1 TK^−^(ACV^r^), parainfluenza-3 virus (PiV), reovirus-1 (RV), sindbis virus (SV), coxsackie virus B4 (CV), Punta Toro virus (PTV), and respiratory syncytial virus (RSV).

The antiviral activity of the above-reported compounds was tested* in vitro* in HEL, HeLa, and Vero cells cultures, where appropriate, along with the reference antiviral compounds such as brivudine, ribavirin, acyclovir, ganciclovir, and (S)-DHPA. Results are, respectively, shown in Tables 2–4.

All synthesized compounds showed no specific antiviral effects; some differences between the regioisomeric nucleosides can be however noted. Regioisomers of type** b**, involving a phenyl group distal to the heterocyclic base, display better response than regioisomers of type** a**, which have proximal phenyl and heterocyclic base. If this indication will be confirmed in other cases, future syntheses will benefit of new efforts to orientate more regioselectively the preparation of new nucleosidic targets. Moreover, all compounds were evaluated for antiviral activity against HIV-1 (strain III_B_) and HIV-2 (strain Rob) in MT-4 cell culture and none of these compounds showed inhibitory activity at concentration up to 400 *μ*g/mL.

The modest antiviral activity showed from these compounds could be likely linked to the lack of substrate activity for cellular and/or viral nucleoside kinases or alternatively the lack of recognition of the compounds by the viral DNA or RNA polymerases.

## 3. Conclusions

In conclusion, the synthesis of isoxazoline-carbocyclic nucleosides was properly tuned as well as improved and a variety of analogues were attained starting from the stereodefined heterocyclic aminols** 11a**,**b**. These latter ones are readily available through* exo* selective 1,3-dipolar cycloadditions of benzonitrile oxide to* N*-benzoyl-oxazanorbornene **8** and simple elaborations of the cycloadducts** 9a**,**b**. The stereodefined heterocyclic aminols** 11a**,**b** afford the carbocyclic skeleton for the linear construction of the purine type, uracil, and thymine moieties.

Compounds** 14a**,**b** and** 16a**,**b** were evaluated for their inhibitory activity against a wide variety of viruses through* in vitro* tests in HEL, HeLa, and Vero cells [32]. Modest antiviral activities were observed. In order to have more insights on the SAR [33] and to increase the antiviral activities of this class of nor-nucleosides their transformation into the corresponding nucleotide monophosphate analogue by esterification of secondary hydroxyl group into phosphonomethyl ether (=P (OH)_2_–CH_2_–O–), which is isosteric of phosphate group (=P (OH)_2_–O–CH_2_–), will be further performed [34].

The biological tests of the thio-derivatives are currently under evaluation. In view of the results obtained with the amino-derivatives, different targets for both the typologies of compounds have been chosen and the results will be accounted in future papers.

## 4. Experimental

All melting points are uncorrected. IR spectra (Nujol mulls) were recorded on a FT-IR Perkin-Elmer RX-1. ^1^H- and ^13^C-NMR spectra were recorded on a Bruker AVANCE 300 in the specified deuterated solvents. Chemical shifts are expressed in ppm from internal tetramethylsilane (*δ*). UV-Vis spectra were recorded on a UV Perkin-Elmer LAMBDA 16 spectrophotometer using acetonitrile as solvent. HPLC analyses were carried out by means of a WATERS 1525 instrument equipped with UV 2487 detector (*λ* = 266 nm) both controlled by Breeze software and a RP C-18 Inertsil ODS-2 column; a mixture of H_2_O/CH_3_CN 50/50 was used as eluent. Column chromatography and tlc: silica gel 60 (0.063–0.200 mm) (Merck); eluent chloroform or chloroform/methanol 9 : 1. The identification of samples from different experiments was secured by mixed mps and superimposable IR spectra.

*Materials*. Regioisomeric aminols** 11a**,**b** were prepared through previously reported syntheses [19] by applying the modified protocols described in this paper to get the aminols in quantitative yields from the regioisomeric cycloadducts** 9a**,**b**.

### 4.1. Synthesis of the Pyrimidine Derivatives** 12a**,**b**

To aminols** 11a**,**b** (3 g, 13.7 mmol) dissolved in* n*-BuOH (75 mL) and* i*-Pr_2_NEt (8.88 g, 68.7 mmol), 5-amino-4,6-dichloropyrimidine (4.50 g, 27.5 mmol) was added. The mixtures were stirred at room temperature for 1 h and then slowly heated at 117°C with stirring for 48 h. The cooled solutions were evaporated to dryness, taken up in CH_2_Cl_2_, washed with brine, and dried over anhydrous Na_2_SO_4_. The crude residues were then submitted to column chromatography to separate the excess of aminopyrimidine from adducts** 12a**,**b** which were isolated in 79% yield for both regioisomeric compounds. Yields improvements are around 30% with respect to previous method. The analytical and spectroscopic data are in full accordance with those reported in [13].

### 4.2. Construction of the Purine Nucleosides** 13a**,**b**

A solution of pyrimidine derivatives** 12a**,**b** (3.50 g, 9.8 mmol) in triethyl orthoformate (30 mL) is stirred at room temperature until the starting material has been completely formulated (TLC monitoring) usually in 3-4 h. A catalytic amount of* p*TsOH was added afterwards and the reaction was stirred at room temperature for 5 days. After this period of time, the excess orthoformate was hydrolyzed in 24 h by adding water and the water phase extracted with CH_2_Cl_2_ and dried over anhydrous Na_2_SO_4_. After evaporation of the solvent, the residue was taken up with diisopropyl ether and few drops of ethyl acetate and the chloropurine derivatives** 13a**,**b** crystallized. Yields improvements are around 10% with respect to previous method. The analytical and spectroscopic data are in full accordance with those previously reported [13].

### 4.3. Syntheses of the Adenine Derivatives** 14a**,**b**(**A**–**D**)

Solutions of chloro-nucleosides** 13a**,**b** (50 mg, 0.14 mmol) in MeOH (3 mL) were saturated with ammonia or other gaseous amines and kept in a sealed tube at 50°C for 24 h. In the case of liquid amines, an excess (40 equiv.) was added to the solutions. The solutions are then cooled and concentration of the solutions afforded oily residues from which the amino derivatives were crystallized from proper solvents. The yields of the amino nucleosides** 14a**,**b**(**A**–**D**) were determined by hplc analyses and are generally excellent (92–99%). The hplc analyses were performed according to the reported conditions by injecting 5 *μ*L of the alcoholic solutions after cooling them to ambient and using external standard method for quantitative determinations [13].

### 4.4. Syntheses of the Thio-Derivatives** 14a**,**bS**

The chloro-nucleosides** 13a**,**b** (25 mg, 0.07 mmol) were dissolved in 2 mL of a 1 : 2 EtOH : H_2_O solution. Na_2_S (13 mg, 0.23 mmol) was added and the mixtures were heated at reflux for 2 h. After this period of time, the pH is adjusted at neutrality upon treatment with a 10% acetic acid solution. Upon cooling, white crystals separated off and were filtrated. The NMR spectra reported below refer to the major tautomer** II**.

**14aS**, white crystals from ethanol, m.p. 205-206°C (dec.). IR (cm^−1^): *ν*_OH_ 3314, *ν*_C=N_ 1607, 1591. ^1^H-NMR (*δ*, CD_3_COCD_3_): 2.65 (m, 2H, CH_2_); 4.54 (dd,* J* 10, 3 Hz, 1H, H4 isox.); 4.64 (m, 3H, CH-OH); 5.31 (m, 1H, CH-N); 5.81 (dd,* J* 10, 3 Hz, 1H, 5 isox.); 7.45 (m, 3H, arom.); 7.85 (m, 2H, arom.); 8.78 (s, 1H, CH=N); 8.83 (s, 1H, CH=N). ^13^C-NMR (*δ*, CD_3_COCD_3_): 38.5, 61.7, 62.0, 75.2, 89.7, 127.1, 128.7, 128.8, 130.0, 139.7, 146.0, 151.3, 157.0, 172.5. Elemental analysis: calcd. For C_17_H_15_N_5_O_2_S (MW = 353.33) C, 57.78; H, 4.28; N, 19.82. Found: C, 57.8; H, 4.3; N, 20.0.

**14bS**, white crystals from ethanol, m.p. 208–210°C (dec.). IR (cm^−1^): *ν*_OH_ 3284, *ν*_C=N_ 1607, 1590. ^1^H-NMR (*δ*, CD_3_COCD_3_): 2.58 (m, 2H, CH_2_); 4.50 (dd,* J* 10, 3 Hz, 1H, H4 isox.); 4.62 (m, 3H,* CH*-OH); 5.25 (m, 1H, CH-N); 5.76 (dd,* J* 10, 3 Hz, 1H, 5 isox.); 7.45 (m, 3H, arom.); 7.85 (m, 2H, arom.); 8.75 (s, 1H, CH=N); 8.78 (s, 1H, CH=N). ^13^C-NMR (*δ*, CD_3_COCD_3_): 37.3, 60.5, 60.8, 74.0, 88.5, 125.9, 127.6, 127.7, 128.8, 142.0, 144.8, 150.1, 155.9, 171.5. Elemental analysis: calcd. For C_17_H_15_N_5_O_2_S (MW = 353.33) C, 57.78; H, 4.28; N, 19.82. Found: C, 57.7; H, 4.2; N, 19.9.

### 4.5. Syntheses of the Isocyanate Adducts** 15a**,**b**

To solutions of aminols** 11a**,**b** (3 g, 13.7 mmol) in anhydrous DMF (15 mL) at –20°C, solutions of the proper isocyanates** U**,**T** (16.4 mmol) in anhydrous benzene were added dropwise with stirring in anhydrous nitrogen atmosphere and in the presence of 6 MS 4 Å. After keeping for one night (12 h) at r.t., the solutions were filtered and solvent was removed under reduced pressure. The residues were submitted to column chromatography to isolate the compounds** 15a**,**b** in 82% yields and purified through crystallization from ethanol. The analytical and spectroscopic data are in full accordance with those previously reported [13].

### 4.6. Construction of the Uracil and Thymine Nucleosides** 16a**,**b**

Adducts** 15a**,**b**(**U**,**T**) (4 mmol) are suspended in 2 M H_2_SO_4_ (25 mL) solutions and gently refluxed for 3 h. After cooling, the pH is adjusted to 7 with NaHCO_3_ and the water phase extracted with dichloromethane. Evaporation of the dried organic phase afforded the uracil or thymine nucleosides** 16a**,**b**(**U**,**T**) (91–95%, +20%) which were purified by simple crystallization.

### 4.7. Antiviral Activity and Cytotoxicity

The synthesized compounds were evaluated for their antiviral and cytotoxicity activity against herpes simplex virus type 1 (HSV-1, strain KOS), herpes simplex virus type 2 (HSV-2, strain G), vaccinia virus (VV), vesicular stomatitis virus (VSV), HSV-1 (KOS) thymidine kinase-deficient (TK^−^) ACV^r^ in HEL cell cultures, VSV, Coxsackie (CV) virus B4 and respiratory syncytial virus (RSV) in HeLa cell cultures, parainfluenza-3 virus (PiV), reovirus-1 (RV), sindbis virus (SV), CV, and Punta Toro virus (PTV) in Vero cells cultures.

The antiviral activity was monitored by plaque reduction. The compounds were first dissolved in 100% DMSO and then diluted with EMEM-5 (Eagle's minimal essential medium) before use. The maximum final concentration of DMSO added to the cell cultures was 0.5% at the highest concentration of the compound and does not interfere with cell growth. The appropriate cells were seeded in 96-well plates at a density of 2 × 10^5^ cells/well and were allowed to form confluent monolayers by incubating overnight in growth medium at 37°C with 5% CO_2_ atmosphere. Monolayers were then inoculated with 100 CCID50 (1 CCID50 being the virus dose required to infect 50% of the cell cultures). After 1 hour of virus adsorption, residual virus was removed and replaced by cell culture medium (Eagle's minimal essential medium) containing various concentrations (400, 200, 100, and 50 *μ*g) of the test compounds. Cultures were incubated at 37°C for 48 h and then strained with 0.1% crystal violet solution. Plaques were then enumerated and the IC_50_ values for the various drugs were determined. The activities of compounds** 14a**,**b**–**16a**,**b** were compared with those of brivudine, ribavirin acyclovir, and ganciclovir.

The cytotoxicity of all compounds was also evaluated. Briefly, HEL, HeLa, and Vero cells were seeded in 96-well plates; 24 h later increasing concentrations of the tested compounds were added to each well. After a further incubation of 24 h at 37°C in a humidified, 5% CO_2_ atmosphere, the cells viability was evaluated by a commercial assay (CellTiter 96 Aqueous One Solution Assay, Promega Co., Madison WI), following the manufacturer's instructions. This assay is based on the principle that cells, at death, rapidly lose the ability to reduce MTS tetrazolium. At the end of the incubation time, the MTS-tetrazolium-based reagent was added to each well. After a further incubation of 1 h (37°C, 5% CO_2_ atmosphere), the absorbance of the samples was recorded at 490 nm using a 96-well spectrophotometer. The cytotoxic concentration 50% was calculated as the concentrations of the compounds** 14a**,**b**–**16a**,**b** required to cause 50% reduction of absorbance values. The antiviral and cytotoxicity data are reported in Tables 2, 3, and 4 [35].

## Figures and Tables

**Table 1:** Tautomeric ratios and chemical shifts for compounds **14a,bS**.

Solvent	I/II	CH=N	Ha	Hb	Hc	Hd	He
		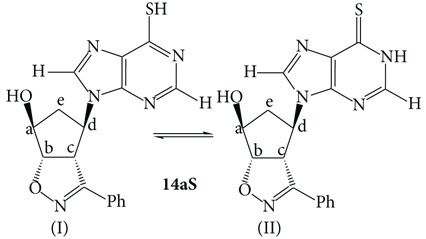					
CD_3_COCD_3_	1/1.5	(I) 8.23, 8.44	4.61	5.73	4.49	5.18	2.59
		(II) 8.78, 8.83	4.64	5.81	4.54	5.31	2.65
DMSO-d6	4/1	(I) 8.14, 8.43	4.30	5.62	4.26	5.00	2.38
		(II) 8.80, 8.81	4.30	5.74	4.26	5.19	2.45
CD_3_OD	1/1	(I) 8.15, 8.50	4.90	5.60	4.45	5.16	2.45
		(II) 8.78, 8.82	4.90	5.75	4.40	5.27	2.45

		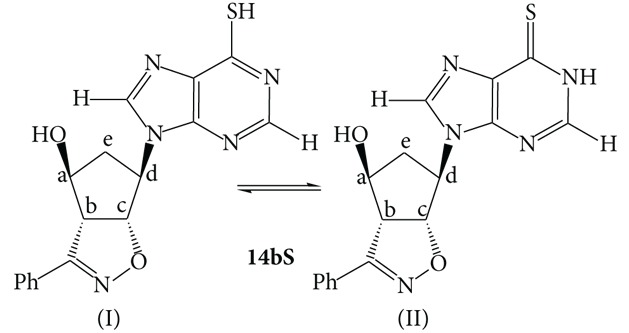					
CD_3_COCD_3_	2/1	(I) 8.25, 8.34	4.62	5.75	4.45	5.15	2.50
		(II) 8.75, 8.78	4.62	5.76	4.50	5.25	2.58
DMSO-d6	1/1	(I) 8.84, 8.87	4.34	5.71	4.34	5.11	2.41
		(II) 8.24, 8.48	4.34	5.61	4.34	5.95	2.32
CD_3_OD	1/1.5	(I) 8.81, 8.85	4.48	5.76	4.48	5.29	2.50
		(II) 8.18, 8.50	4.48	5.67	4.48	5.16	2.50

**Table 2:** Cytotoxicity and antiviral activity of compounds **14a,b** and **16a,b** in HEL cell cultures.

Compound	MCC^a^ (*μ*g/mL)	MIC^b^ (*μ*g/mL)				
		HSV-1 (KOS)	HSV-2 (G)	VV	VSV	HSV-1 TK-ACV^r^
**14aA**	80	>16	>16	>16	>16	>16
**14aB**	>400	>400	>400	>400	>400	>400
**14aC**	80	>16	>16	>16	>16	>16
**14aD**	400	>80	>80	>80	>80	>80
**14bA**	16	>3.2	>3.2	>3.2	>3.2	>3.2
**14bB**	>400	>400	>400	>400	>400	>400
**14bC**	80	>16	>16	>16	>16	>16
**14bD**	16	>3.2	>3.2	>3.2	>3.2	>3.2
**16aU**	>400	>400	>400	>400	>400	>400
**16aT**	>400	>400	>400	>400	>400	>400
**16bU**	400	>80	>80	>80	>80	>80
**16bT**	400	>80	>80	>80	>80	>80

Brivudine	>400	0.128	400	48	>400	>400
Ribavirin	>400	>400	48	240	>400	>400
Acyclovir	>400	0.64	0.64	>400	>400	400
Ganciclovir	>400	0.096	0.16	>100	>100	12

^a^Minimum cytotoxic concentration, required to cause a microscopically detectable alteration of normal cell morphology.

^b^Minimum inhibitory concentration required to reduce virus-induced cytopathogenicity by 50%.

**Table 3:** Cytotoxicity and antiviral activity of compounds **14a,b** and **16a,b** in HeLa cell cultures.

Compound	MCC^a^ (*μ*g/mL)	MIC^b^ (*μ*g/mL)		
		VSV	CV	RSV
**14aA**	80	>16	>16	>16
**14aB**	400	>80	>80	>80
**14aC**	≥16	>16	>16	>16
**14aD**	80	>16	>16	>16
**14bA**	≥80	>80	>80	>80
**14bB**	≥80	>80	>80	>80
**14bC**	≥16	>16	>16	>16
**14bD**	≥80	>80	>80	>80
**16aU**	400	>80	>80	>80
**16aT**	≥80	>80	>80	>80
**16bU**	400	>80	>80	>80
**16bT**	≥80	>80	>80	>80

Brivudin	>400	>400	>400	>400
Ribavirin	>400	48	80	48
(S)-DHPA	>400	>400	>400	>400

^a^Minimum cytotoxic concentration, required to cause a microscopically detectable alteration of normal cell morphology.

^b^Minimum inhibitory concentration required to reduce virus-induced cytopathogenicity by 50%.

**Table 4:** Cytotoxicity and antiviral activity of compounds **14a,b** and **16a,b** in Vero cell cultures.

Compound	MCC^a^ (*μ*g/mL)	MIC^b^ (*μ*g/mL)				
		PiV	RV	SV	CV	PTV
**14aA**	80	>16	>16	>16	>16	>16
**14aB**	400	>80	>80	>80	>80	>80
**14aC**	80	>16	>16	>16	>16	>16
**14aD**	400	>80	>80	>80	>80	>80
**14bA**	≥400	>80	>80	>80	>80	>80
**14bB**	400	>80	>80	>80	>80	>80
**14bC**	80	>16	>16	>16	>16	>16
**14bD**	400	>80	>80	>80	>80	>80
**16aU**	400	>80	>80	>80	>80	>80
**16aT**	400	>80	>80	>80	>80	>80
**16bU**	400	>80	>80	>80	>80	>80
**16bT**	400	>80	>80	>80	>80	>80

Brivudine	>400	>400	>400	>400	>400	>400
Ribavirin	>400	48	48	240	48	48
(S)-DHPA	>400	48	240	>400	>400	>400

^a^Minimum cytotoxic concentration, required to cause a microscopically detectable alteration of normal cell morphology.

^b^Minimum inhibitory concentration required to reduce virus-induced cytopathogenicity by 50%.
